# Assessment of Protective Effects of DTPA, NAC, and Taurine on Possible Cytotoxicity Induced by Individual and Combined Zinc Oxide and Copper Oxide Nanoparticles in SH-SY5Y Cells

**DOI:** 10.1007/s12011-024-04161-0

**Published:** 2024-04-29

**Authors:** Duygu Paslı, Aylin Gürbay

**Affiliations:** Department of Pharmaceutical Toxicology, Faculty of Pharmacy, Hacettepe University, 06100 Ankara, Turkey

**Keywords:** Zinc oxide nanoparticles, Copper oxide nanoparticles, Cytotoxicity, Mixture toxicity, Protective agents, Human neuroblastoma cells

## Abstract

The present study investigated the cytotoxic effects of ZnO, CuO, and mixed combinations of them on SH-SY5Y cells. For this purpose, the cells were exposed to various concentrations of these NPs alone for 24–96 h and as a mixture for 24 h. Variations in cell viability were noted. MTT results showed that ZnO and/or CuO NPs decreased cell survival by about 59% at 200 (ZnO, at 24 h) and 800 µg/ml (ZnO and/or CuO, at 72 and 96 h). When the NR assay was used, slight decreases were noted with ZnO NPs at 72 and 96 h. With CuO NPs alone and NPs in a mixture, only the highest concentrations caused 40 and 70% decreases in cell survival, respectively. Especially with NR assays, DTPA, NAC, or taurine provided marked protection. ROS levels were increased with the highest concentration of CuO NPs and with all concentrations of the mixture. The highest concentration of ZnO NPs and the lowest concentration of CuO NPs caused slight decreases in mitochondrial membrane potential levels. Additionally, increases were noted in caspase 3/7 levels with ZnO and CuO NPs alone or with a mixture of them. Intracellular calcium levels were decreased in this system. These findings demonstrated that ZnO and CuO NPs, either separately or in combination, had a modest cytotoxic effect on SH-SY5Y cells. Protection obtained with DTPA, NAC, or taurine against the cytotoxicity of these NPs and the ROS-inducing effect of CuO NPs and the NPs’ mixture suggests that oxidative stress might be involved in the cytotoxicity mechanisms of these NPs.

## Introduction

Exposure to human-made nanoparticles (NPs) including metal NPs is increasing rapidly in today’s world [1–4]. Considering the lack of exact information on their toxic effects, more studies are needed to show their possible unwanted effects in different model systems. On the other hand, organisms might be exposed to NPs as a mixture. As mixture studies are gaining importance in the toxicology area for years [5], investigations on the toxicity of a mixture of man-made NPs are also important.

The effects of man-made NPs on brain health is also a crucial research field, and available data suggest that the blood–brain barrier (BBB) cannot provide effective protection against these types of NPs [6]. Regarding the very special role, complex functions, and importance of the brain in the human organism on the one hand, and numerous unanswered questions on the toxicity of man-made NPs in different model systems on the other, there are raising concerns about potentially toxic effects of man-made NPs on the brain [6, 7].

ZnO and CuO NPs with their special properties are examples of the widely used metal NPs [8]. Their antiviral, antifungal, and antibacterial properties make them preferred among other NPs [8–10]. For instance, ZnO NPs are used in drug delivery, the pharmaceutical industry, and biomedical engineering, as well as in sunscreens, food additives, dental fillings, and cosmetics. And, CuO NPs are wielding in numerous biomedical applications, magnetic storage media, and the production of sensors, semiconductors, biosensors, and near-infrared filters [9–15]. As a result of their increasing usage, interaction possibilities between organisms and these two NPs are also rising [10, 16]. Additionally, there is not enough study regarding health risks due to exposure to a mixture of NPs. In a study performed by Parsai and Kumar [17], they indicate there is a lack of data on the interaction of toxicity of ZnO and CuO NPs. And in order to estimate the reference dose, and decide synergism and antagonism effects of these NPs/ion combinations, further information on the toxicity of these NPs is needed [17].

There are numerous *in vitro* studies on the cytotoxic effects of manufactured NPs in different central nervous system cells [18–20]. However, investigations on the cytotoxic effects of ZnO and CuO NPs are limited [4, 21–26].

Regarding the above-mentioned information, this study aims to determine the possible time- and dose-dependent cytotoxic effects of ZnO and CuO NPs alone or as a mixture on human neuroblastoma (SH-SY5Y) cells. The protective effects of diethylenetriaminepentaacetic acid (DTPA), N-acetylcysteine (NAC), and taurine against NPs-induced toxicity were also examined in the same model system. To the best of our knowledge, this is the first study to examine the toxic effects of mixtures of ZnO (< 100 nm) and CuO NPs (< 50 nm) on SH-SY5Y cells.

## Materials and Methods

### Chemicals

ZnO NPs (< 100 nm) and CuO NPs (< 50 nm) were obtained from Sigma-Aldrich (Missouri, USA). The cell culture solutions were from Biological Industries (Kibbutz Beit-Haemek, Israel) and Biowest (Nuaillé, France). Plastic culture materials were purchased from Corning (New York, USA) and Greiner (Kremsmünster, Austria). The other chemicals were obtained either from Sigma-Aldrich (Missouri, USA) or Merck (Darmstadt, Germany). 5,5′,6,6′-Tetrachloro-1,1′,3,3′-tetraethylbenzimidazolyl-carbocyanine iodide derivative (JC-10) and carbonylcyanide-4-(trifluoromethoxy)-phenylhydrazone (FCCP) were from Enzo Life Sciences (Farmingdale, USA).

### Preparation and Characterization of Nanoparticle Solutions

Stock solutions of ZnO (< 100 nm, Sigma) and CuO (< 50 nm, Sigma) NPs were prepared by dispersing the NPs at 10 mg/ml concentrations in pure water. Solutions were sonicated for 15 min at 35 kHz (Elma Transsonic 460/H, Germany) separately for dispersing the particles. Then, the solutions were sterilized using a 0.20-μm pore-sized sterile filter. Standard solutions were prepared from stock solutions using a culture medium. All stock and standard solutions were freshly prepared each day. FEI Tecnai G2 Spirit Bio (TWIN) High Contrast Transmission Electron Microscope (Hillsboro, USA) was used to evaluate the shape and size of ZnO and CuO NPs. For a 1-day drying process, nanoparticle solutions were dropped on a copper grid covered with 200 mesh carbon film. Then, transmission electron microscope (TEM) images of these nanoparticles were obtained at 120 kV.

### Cell Culture

The human neuroblastoma cell line was purchased from the American Type Culture Collection (ATCC, USA). The cell line was cultured in 10% fetal calf serum-rich DMEM: F-12 (Ham’s) medium supplemented with 1% L-glutamine and 1% penicillin–streptomycin at 37 °C in a humidified atmosphere containing 5% CO_2_ and 95% air in 75 cm^2^ plastic culture flasks. For all methods tested, dissociated cells were seeded in 96-well culture plates. To allow adherence and proliferation, cells were incubated for 24 h at 37 °C in a 5% CO_2_ humidified atmosphere. When the cultures reached 95% confluence, they were used in the assays as explained below.

### Cytotoxicity Studies

For cytotoxicity studies, medium containing ZnO (0.1 to 800 μg/ml) or CuO NPs (0.01 to 800 μg/ml) were added to the wells. Then, the cell cultures were incubated for 24, 48, 72, or 96 h at 37 °C. Potential mitochondrial and lysosomal cytotoxic effects were determined with MTT and NR assays described by Mosmann [27] and Borenfreund and Puerner [28], respectively. For combined NPs, 24- and 48-h incubation times were chosen for MTT and NR assays, respectively. Protective effects of DTPA, NAC, or taurine were also evaluated in this model system.

### Determination of Reactive Oxygen Species

To determine the possible formation of reactive oxygen species (ROS), a method described by Cathcart et al. [29] was applied to cell cultures using a fluorescent probe, DCFH-DA. For this purpose, SH-SY5Y cells were exposed to ZnO (100, 400, 800 μg/ml) or CuO NPs (100, 400, 800 μg/ml) alone or as a mixture (100:100, 400:400, 800:800 μg/ml) in 96-well clear bottom black plates and incubated for 24 h. Then, DCFH-DA solution (10 µM) was added to each well, and cell cultures were incubated in the dark at 37 °C for 45 min. Tert-butyl hydroperoxide (TBHP) (15 µM) was used as a positive control. Fluorescence was measured at 485 nm excitation and 535 nm emission by using a fluorescent microplate reader (SpectraMax, San Jose, USA). The percentage of control fluorescence was calculated for each result.

### Mitochondrial Membrane Potential

Mitochondrial membrane potential (MMP) was determined using a cationic dye, JC-10 [30–32]. Cells in 96-well clear bottom black plates were exposed to ZnO (100, 400, 800 μg/ml) or CuO NPs (100, 400, 800 μg/ml) alone or as a mixture (100:100, 400:400, 800:800 μg/ml) for 24 h. Then, the cells were incubated with JC-10 dye (1 µM) in the dark at 37 °C for 20 min. FCCP (1 µM) was used as a positive control for depolarizing mitochondrial membrane potential. Red and green fluorescence of cells were measured at 540 nm excitation, 590 nm emission, and at 490 nm excitation, 525 nm emission, respectively, by using a fluorescent microplate reader (SpectraMax, San Jose, USA). The red/green fluorescence ratio was calculated to obtain change of mitochondrial membrane potential. Results were given as a percentage of the control.

### Apoptosis and Intracellular Calcium Levels

#### Preparation of Cells for Apoptosis and Intracellular Calcium Levels

SH-SY5Y cells were seeded in 96-well clear bottom black plates and were incubated for 24 h at 37 °C in a 5% CO_2_ humidified atmosphere to allow adherence and proliferation. Then, the cell cultures were exposed to 100 and 800 µg/ml concentrations of ZnO, and CuO NPs alone or as a mixture following preincubation of cells with protective agents for 4 h. Possible protective effects of DTPA (5 μg/ml), NAC (1 mM), and taurine (1 mM) were evaluated against 100 µg/ml concentrations of ZnO and/or CuO NPs. Details of apoptosis and intracellular calcium levels assays were described below:

#### Apoptosis

To determine cellular apoptosis, ApoOne Homogeneous Caspase-3/7 Assay Kit (Promega, Wisconsin, USA) was used. The kit procedure was applied to the cell cultures as described. Then, the plates were incubated in the dark at room temperature for 30 min. Fluorescence levels were determined at 485 nm excitation and 528 nm emission wavelength. These levels were compared with control fluorescence levels for the presentation of results.

#### Intracellular Calcium Levels

A Fluoforte Calcium Assay Kit (Enzo Life Sciences, Farmingdale, USA) was used to evaluate intracellular calcium levels. Following the incubation of cell cultures with NPs, Fluoforte dye was added to each well, and plates were incubated in a dark place at 37 °C for 45 min. Fluorescence was measured at an excitation of 490 nm and an emission of 525 nm.

### Statistical Analysis

Mann–Whitney *U* test was employed to calculate the statistical significance between control and treated groups. A *p*-value < 0.05 was considered to be statistically significant. All data were expressed as mean ± standard deviation, and at least three separate experiments were performed for each method.

## Results

### TEM Images of Zinc Oxide and Copper Oxide Nanoparticles

**Fig. 1:**
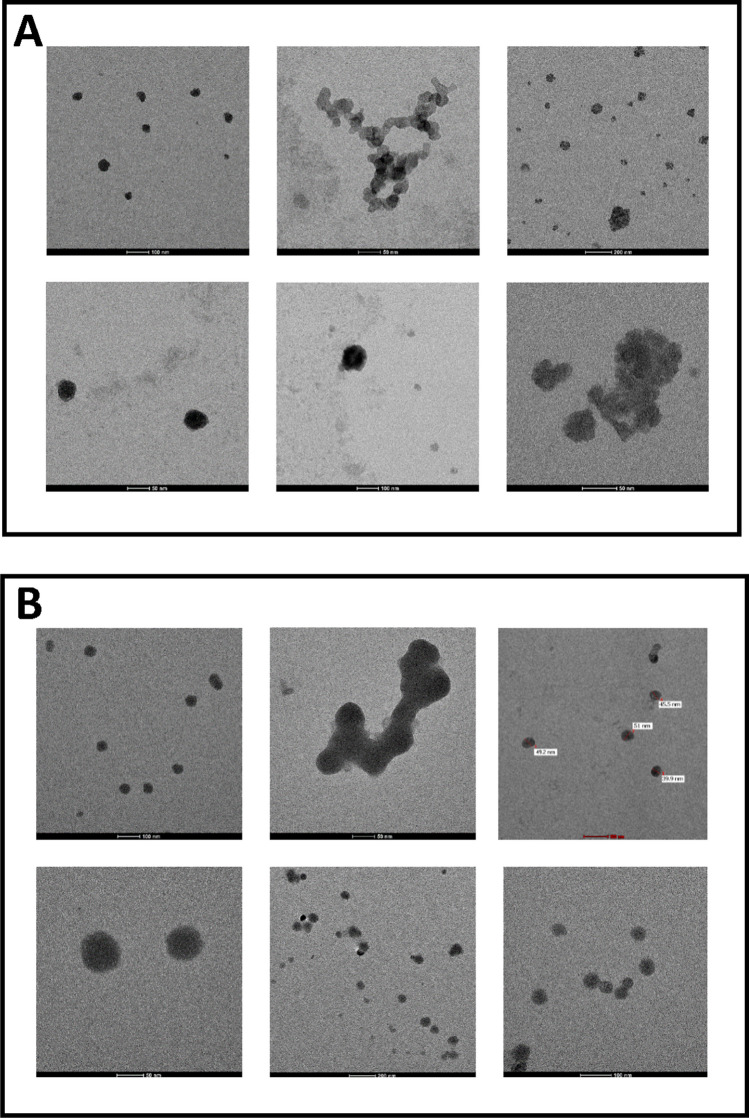
TEM images of (**A)** ZnO and (**B)** CuO NPs dispersed in pure water at 10 mg/ml. Dimensions of both nanoparticles were found appropriate to the sizes specified on the packages

Transmission electron microscope images of ZnO and CuO NPs show sizes and agglomeration or aggregation states of stock solutions of these NPs (Fig. 1). Dimensions of both nanoparticles were appropriate to the sizes specified on the packages (ZnO < 100 nm, CuO < 50 nm).

### Cytotoxicity

#### Mitochondrial Cytotoxicity

**Fig. 2:**
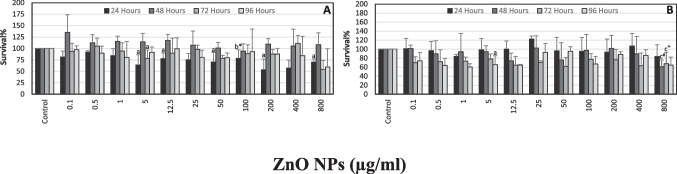
The effect of ZnO NPs on SH-SY5Y cell survival following 24, 48, 72, and 96 h. MTT (**A**) and NR (**B**) assays were used to determine survival%. For each concentration in each plate, values are the mean ± S.D. of three to four separate experiments assessed in four or eight replicates. *n = 12, ^#^*n* = 9, ^+^*n* = 13. ^a^*p* < 0.04 vs. control, ^b^*p* < 0.002 vs. control, ^c^*p* < 0.02 vs. control

MTT results of ZnO NPs are represented in Fig. 2A. Slight decreases in cell survival were noted following 24-, 72-, or 96-h incubations. However, no cytotoxic effects were observed at 48 h. Cell viability was ≤ 57% following 24-h incubation at 200 and 400 μg/ml. Similarly, following incubation with ZnO NPs for 72 or 96 h, cell viability was determined as 54.06 and 59.62% at 800 μg/ml, respectively.

**Fig. 3:**
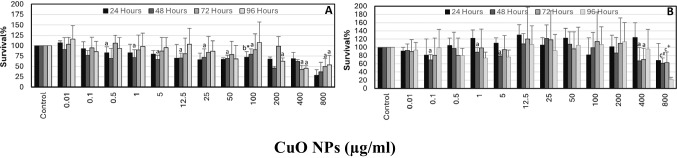
The effect of CuO NPs on SH-SY5Y cell survival following 24, 48, 72, and 96 h. MTT (**A**) and NR (**B**) assays were used to determine survival%. For each concentration in each plate, values are the mean ± S.D. of three to five separate experiments assessed in four or eight replicates. *n = 12, ^#^*n* = 8, ^+^*n* = 13. ^a^*p* < 0.04 vs. control, ^b^*p* < 0.002 vs. control, ^c^*p* *<*0.02 vs. control

There was a slight dose-dependent decrease in percent survival following 24-h incubation of SH-SY5Y cells with various concentrations of CuO NPs (Fig. 3A). The concentrations of CuO NPs which caused < 50% decrease in cell viabilities were 800 μg/ml, 200 and 800 μg/ml, 400 and 800 μg/ml, and 400 μg/ml for 24, 48, 72, and 96 h, respectively.

**Fig. 4:**
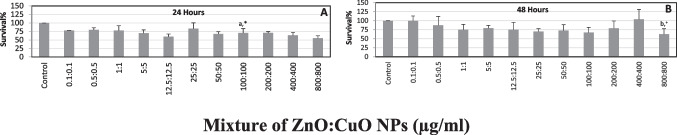
The effect of ZnO:CuO NPs on SH-SY5Y cell survival. MTT (**A**) and NR (**B**) assays were used to determine survival%. For each concentration in each plate, values are the mean ± S.D. of three separate experiments assessed in four or eight replicates. *n = 12, ^+^*n* = 8. ^a^*p* < 0.002 vs. control, ^b^*p* < 0.02 vs. control

When the cells were exposed to the mixture of ZnO: CuO NPs, the highest decreases in cell viability were measured following incubation with 12.5:12.5 and 800:800 μg/ml. The cell viability levels were determined as 59.62% and 54.93%, respectively, for those concentrations (Fig. 4).

**Fig. 5:**
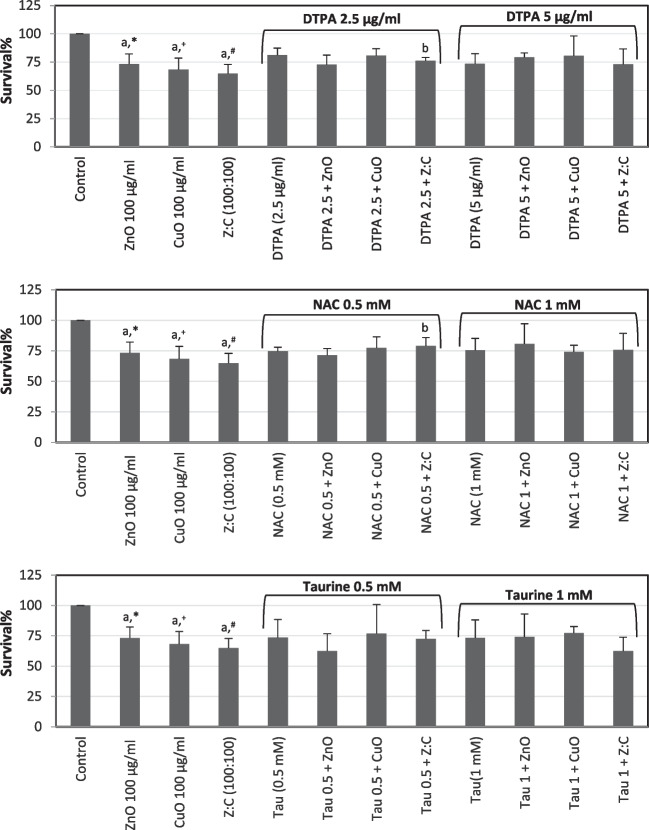
The protective effects of two different concentrations of DTPA, NAC, and taurine on individual and combined ZnO, CuO NPs-induced cytotoxicity in SH-SY5Y cells. The cells were preincubated with protective agents for 4 h before addition of 100 μg/ml NPs for 24 h. MTT assay was used to determine survival%. For each concentration in each plate, values are the mean ± S.D. of three to four separate experiments assessed in four or ten replicates. *n = 8, ^+^*n* = 10, ^#^*n* = 9. ^a^*p* < 0.002 vs. control, ^b^*p* < 0.05 vs. ZnO:CuO (100:100)

Slight protective effects were obtained following preincubation of cells with DTPA, NAC, or taurine against ZnO-, CuO-, or mixture-induced cytotoxicity (Fig. 5).

#### Lysosomal Cytotoxicity

Neutral red assay results of ZnO NPs were shown in Fig. 2B. No cytotoxic effects were observed in SH-SY5Y cells following 24-h incubation with these NPs. There were slight fluctuations in cell viability following 48-h incubation, and the lowest cell survival percent was determined as 61.32% at 800 µg/ml of ZnO NPs. A general 30% decline was observed in the survival percent of cells following 72 h of incubation, and the lowest values determined were 64.45, 61.97, and 63.07 at 12.5, 50, and 400 µg/ml of ZnO NPs, respectively. Cell viability was < 70% at concentrations of 0.5–12.5 µg/ml and 100 and 800 µg/ml following 96-h incubation.

Results of NR assay in the presence of CuO NPs showed that (Fig. 3B) only the highest concentrations of these NPs caused marked decreases (nearly 40%) in cell survival at all incubation times especially following 96-h incubation. The determined percent survival at 96 h was 22.38%.

Neutral red assay results of a mixture of ZnO and CuO NPs were given in Fig. 4. Following 48 of hours incubation, < 70% cell survival was noted only with 100:100 or 800:800 µg/ml of ZnO:CuO NPs.

**Fig. 6:**
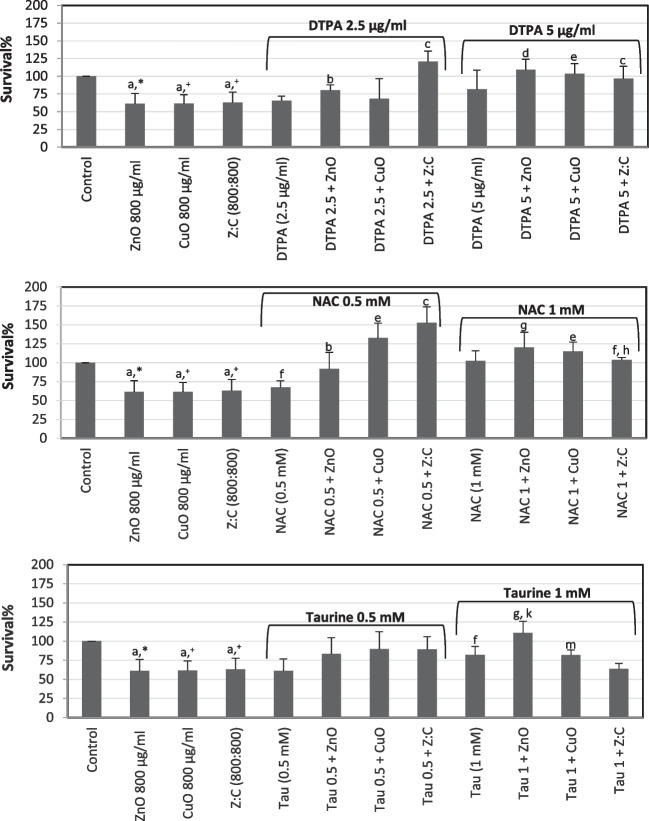
The protective effects of two different concentrations of DTPA, NAC, and taurine on individual and combined ZnO, CuO NPs-induced cytotoxicity in SH-SY5Y cells. The cells were preincubated with protective agents for 4 h before addition of 800 μg/ml NPs for 48 h. NR assay was used to determine survival%. For each concentration in each plate, values are the mean ± S.D. of three to four separate experiments assessed in four or nine replicates. *n = 9, ^+^*n* = 8. ^a^*p* < 0.02 vs. control, ^b^*p* < 0.05 vs. ZnO, ^c^*p* < 0.02 vs. ZnO:CuO, ^d^*p* < 0.02 vs. ZnO, ^e^*p* < 0.02 vs. CuO, ^f^*p* < 0.05 vs. control, ^g^*p* < 0.01 vs. ZnO, ^h^*p* < 0.01 vs. ZnO:CuO, ^k^*p* < 0.05 vs. Tau, ^m^*p* < 0.05 vs. CuO

**Fig. 7:**
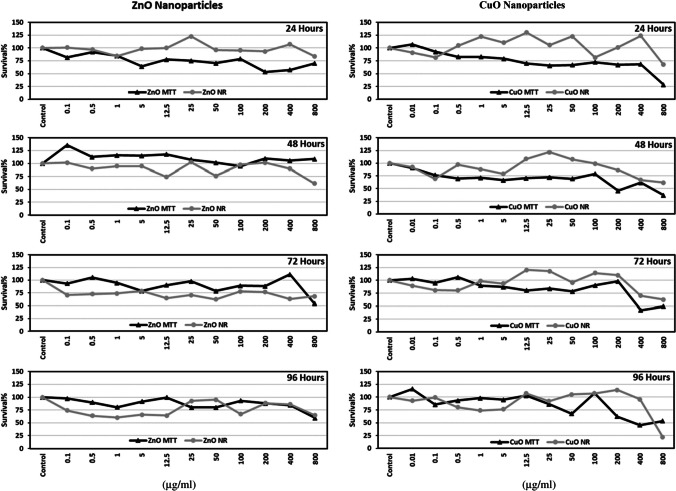
Survival% values of SH-SY5Y cells were determined with MTT and NR assays following 24, 48, 72, or 96 h incubation with ZnO or CuO NPs

Protective effects of DTPA (2.5 and 5 µg/ml), NAC (0.5 and 1 mM), or taurine (0.5 and 1 mM) determined with NR assay were shown in Fig. 6. Preincubation (4 h) of cells with two different concentrations of these agents had marked protective effects on cell viability. Only a higher concentration of taurine (1 mM) did not have any effects on cytotoxicity induced by NP’s mixture. A comparison of both NPs’s cytotoxicity assay results was given in Fig. 7.

### Reactive Oxygen Species Generation

**Fig. 8:**
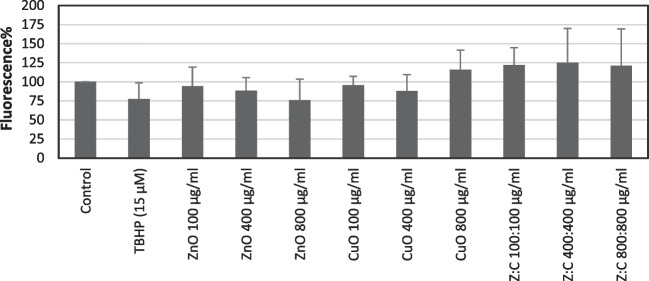
The effects of individual and combined ZnO, CuO NPs on reactive oxygen species generation in SH-SY5Y cells following incubation for 24 h. For each concentration in each plate, values are the mean ± S.D. of three to four separate experiments performed in four or eight replicates. There is no statistically significance when the results compared with control or TBHP

DCFH-DA analysis results were shown in Fig. 8. In this experiment, tert-butyl hydroperoxide (TBHP) (15 µM) was used as a positive control. However, no increase was noted in fluorescence percent following incubation with this agent in the present model system. Similarly, ZnO NPs did not induce ROS production. We observed that only the highest concentration (800 µg/ml) of CuO NPs and all the concentrations of mixture caused increases in fluorescence percent.

### Mitochondrial Membrane Potential

**Fig. 9:**
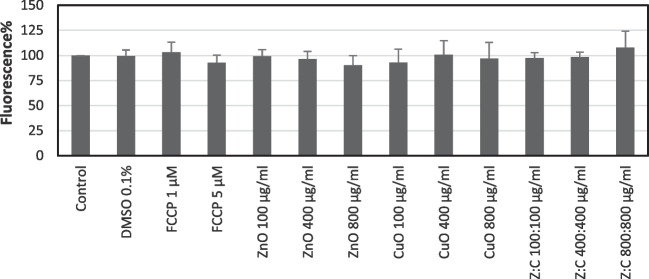
Mitochondrial membrane potential changes in SH-SY5Y cells following incubation with individual and combined ZnO, CuO NPs for 24 h. For each concentration in each plate, values are the mean ± S.D. of three separate experiments performed in four or eight replicates. There is no statistical significance when the results compared with control, DMSO control, or FCCP

In mitochondrial membrane potential assays, FCCP was used as a positive control. In this model system, only higher concentration (5 µM) of FCCP decreased MMP levels. DMSO was used as a solvent, and it has no effect on the SH-SY5Y cell culture. A dose-dependent decline was observed in MMP levels following the incubation of cells with ZnO NPs for 24 h. Slight decreases were noted as 92.96 and 97.15% for 100 and 800 µg/ml concentrations of CuO NPs and 97.43 and 98.53% for 100:100 and 400:400 µg/ml ZnO: CuO NPs, respectively (Fig. 9). There is no statistical significance when the results compared with control, DMSO control, or FCCP.

### Apoptosis

Caspase 3/7 levels were increased following incubation of SH-SY5Y cells with two different concentrations of ZnO and/or CuO NPs, and all treatments with DTPA for 24 h. However, following preincubation of cells with NAC or taurine in the presence or absence of ZnO, CuO, or mixture of them, decreases were noted in caspase 3/7 levels except “ZnO plus NAC” treatment (Table 1).

**Table 1:** The effects of individual and combined ZnO, CuO NPs on caspase 3/7 and intracellular calcium levels in SH-SY5Y cells following incubation for 24 h

Substance	Caspase 3/7 levels (fluorescence)^a^	Intracellular calcium levels (fluorescence%)^b^
Control	169.75 ± 27.88	100.00 ± 0.00
ZnO 100 µg/ml	203.82 ± 69,78	81.98 ± 21.07
CuO 100 µg/ml	219.17 ± 82.99	63.16 ± 16.56
Z:C 100:100 µg/ml	186.55 ± 42.58	61.26 ± 20.75
ZnO 800 µg/ml	282.42 ± 118.28	84.11 ± 19.95
CuO 800 µg/ml	224.50 ± 103.32	77.38 ± 36.36
Z:C 800:800 µg/ml	171.67 ± 11.20	85.32 ± 32.71
DTPA 5 µg/ml	226.44 ± 25.51	57.28 ± 18.12
DTPA 5 µg/ml + Z100 µg/ml	236.49 ± 65.02	55.17 ± 17.77
DTPA 5 µg/ml + C100 µg/ml	222.58 ± 49.00	50.62 ± 4.77
DTPA 5 µg/ml + Z:C100:100 µg/ml	201.84 ± 48.35	57.72 ± 10.72
NAC 1 mM	157.59 ± 34.19	49.86 ± 7.38
NAC 1 mM + Z100 µg/ml	203.62 ± 77.47	58.82 ± 14.10
NAC 1 + C100 µg/ml	108.24 ± 7.42	51.34 ± 9.02
NAC 1 + Z:C100:100 µg/ml	125.61 ± 12.70	72.94 ± 23.10
Tau 1 mM	161.58 ± 20.48	33.66 ± 14.88
Tau1 + Z100 µg/ml	164.80 ± 16.60	63.96 ± 19.53
Tau1 + C100 µg/ml	168.85 ± 35.76	53.18 ± 2.06
Tau1 + Z:C100:100 µg/ml	172.57 ± 38.36	53.00 ± 32.23

^a^For each concentration in each plate, values are the mean ± S.D. of three separate experiments assessed in two or three replicates

^b^For each concentration in each plate, values are the mean ± S.D. of three separate experiments assessed in four or 13 replicates

### Intracellular Calcium Levels

Intracellular calcium levels of cells declined following 24-h exposure to ZnO and/or CuO NPs. The results of protective agents in the presence or absence of NPs also showed similar results (Table 1).

## Discussion

It has been reported that information on the neurotoxic effects of NPs has been incomplete and a continual debate exists on this subject [6]. Considering either the complexity of the nervous system or unknown properties and unexplained effects of human-made NPs in the brain, investigations on the neurotoxic effects of these types of NPs are important. In the present study, using SH-SY5Y cells as a model system for neurons [33], we investigated single or mixed cytotoxic effects of human-made ZnO and CuO NPs.

In the current study, for the preparation of stock solutions of ZnO and CuO NPs, the filter sterilization method was preferred. As indicated, this method is the only option for metal as well as other hard-structured NPs (e.g., polymeric or silica-based NPs) [34]. On the other hand, characterization studies were performed with TEM following the sterilization procedure, and the sizes of both NPs were found compatible with the dimensions stated on the packages. In the literature, aggregates were determined in characterization studies of ZnO and CuO NPs, separately [35–37]. In the present study, agglomeration state and distributions of ZnO and CuO NPs in stock solutions were found similar to literature data.

In the present study, a wide concentration range and four different incubation periods were chosen to obtain mitochondrial and lysosomal cytotoxicity profiles of ZnO and CuO NPs. Then, the cytotoxic effects of these NPs as a mixture were investigated following 24 h of incubation using the same concentration range for ZnO and CuO NPs alone. Concentration ranges were chosen according to previous studies performed with these NPs [22, 36, 38–40].

Our results indicated that ZnO NPs caused slight decreases in the survival percent of cells at 24 h when mitochondrial cytotoxic effects were determined with MTT assay. At this time point, the lowest percent survival we noted was 53% at 200 µg/ml (Fig. 2). Similarly, cytotoxic effects of ZnO NPs in SH-SY5Y cells were also shown by Kim et al. (2015) following 24 h of incubation. As their concentration range was narrow (10–30 µg/ml), we cannot make a comparison for our higher concentrations.

Following 48 h of incubation, different from other studies, we observed that ZnO NPs caused the proliferation of cells almost at all concentrations except 100 µg/ml. This effect might be the result of the induction of defense mechanisms of cells following exposure to these NPs at this time point. However, to make an exact conclusion, measurement of oxidative stress and/or antioxidant levels (e.g., glutathione or activity of antioxidant enzymes) is required. Actually, ROS levels were determined in the present study; however, the incubation time for this assay chosen was 24 h (Fig. 8). Increases in cell proliferation were also shown at ≤ 3 µg/ml of ZnO NPs on human umbilical vein endothelial cells at 5 days by Tsou et al. [41]. In addition, Pan et al. [26] reported that ZnO NPs showed protective as well as cytotoxic functions changing Zn^2+^ concentration and modulating expression of Zn transporter_1_ and Zrt/Irt-related proteins (ZIP)_8_ in SH-SY5Y cell culture. Therefore, the results obtained in our study might be related to this dual role of zinc.

At 72 and 96 h of incubation periods, the lowest percent survival values we determined as 54 and 59%, respectively, only at 800 µg/ml. Our MTT assay results were found different from, for instance, the results of Valdiglesias et al. [23] for these time points. Differences determined from other studies might be due to several factors including sizes and preparation of NP solutions as well as types of assays used.

Considering the lysosomal cytotoxicity results of the current study with ZnO NPs, we showed that these NPs caused slight decreases in cell survival percent, especially at 72 and 96 h of incubation. The lowest percent of survival determined was 60.44% at 1 µg/ml concentration following 96 h of incubation. These results were different from the data of Valdiglesias et al. [23]. They found that ZnO NPs caused a dose- and time-dependent lysosomal cytotoxicity in SH-SY5Y cells (≥ 25 µg/ml of ZnO NPs for 24 and 48 h).

In the present study, it has been shown that slight decreases in survival percent of cells in the presence of ZnO NPs were more noticeable, in general, with NR assay except 24 h compared to MTT assay (Fig. 7). It has been reported that ZnO NPs showed high solubility in acidic environments like lysosomes with pH 5.2 [4, 42], and release zinc ions [4]. Numerous studies also indicated that the toxicity of ZnO NPs is related to released zinc ions [13, 43–46]. Although we did not measure Zn^2+^ ion concentrations in this study, it could be suggested that more pronounced induction of lysosomal cytotoxicity by ZnO NPs might be related to zinc ion release due to the acidic environment of this organelle.

Regarding our MTT results with CuO NPs, dose-dependent slight decreases in cell viability were observed especially at 24 and 48 h. These results were similar to data of Jang et al. [25] and Chen et al. [21]. Lysosomal cytotoxicity results of CuO NPs showed more fluctuations nearly at all concentrations and incubation times. Only at 96 h with the highest concentration of CuO NPs, the lowest survival percent was determined as 22%. On the other hand, we noted that CuO NPs caused increases in cell survival percent at 24 h nearly with all concentrations tested (0.5 to 50 µg/ml and at 400 µg/ml) when the NR test was used. Increases were also observed nearly at all concentrations between 12.5 and 200 µg/ml of CuO NPs following 72 and 96 h of incubation.

On the other hand, current results of two cytotoxicity tests in the presence of CuO NPs showed a different profile: With CuO NPs, mitochondrial cytotoxicity was more pronounced especially at 24 and 48 h as well as ≥ 1 µg/ml at 72 h. Hsueh et al. [47] reported that CuO NPs are more soluble in acidic pH. They suggested that CuO NPs showed higher toxicity due to the increasing release of Cu^2+^ ions. As mitochondrial pH levels are around 8 [48, 49], our above-mentioned explanation for lysosomal cytotoxicity of ZnO NPs cannot be applied to this situation. Therefore, further studies are needed to explain these effects.

Humans and all other organisms are exposed to various man-made nanoparticles and mixtures of them via several routes. Hence, the determination of their toxic effects, especially as a mixture is an important research area. In several studies, the toxic effects of ZnO and CuO NPs’ mixture on plants [50] and aquatic organisms [46, 51] have been shown. This is the first study investigating the cytotoxic effects of ZnO and CuO NPs’ mixture in human cell culture systems.

Preincubation of cells with two different concentrations of DTPA, NAC, or taurine mildly protected cells from ZnO-, CuO-, and the mixture of these NP-induced slight cytotoxicity when the MTT test was used. Protective effects obtained by these agents were much more pronounced with the NR assay. Overall, this protection was found to be particularly significant in the presence of 0.5 mM NAC against cytotoxicity caused by both CuO NPs- and mixture of these NPs. Considering the protection provided by DTPA, it could be suggested that metal ions might be released from ZnO and/or CuO NPs. Since we did not measure metal ion release in the present model system, we cannot draw an exact conclusion on this subject. Kim et al. [9] also showed that ZnO NP-induced ROS production was suppressed by an iron-specific chelator, deferoxamine. They [9] indicated that this finding is important suggesting future use of metal chelators in the therapy or prevention of different diseases caused by metal-associated NPs [9, 13, 52]. Protective effects obtained with a glutathione precursor NAC [24] in the present study suggest that oxidative stress might be involved in the cytotoxicity mechanisms of these NPs. Supporting our results, Kim et al. (2015) also showed the protective effects of NAC against ZnO NPs-induced cytotoxicity in SH-SY5Y cells. With its antioxidant properties [53–56] as well as the capacity to improve mitochondrial function and inhibit the generation of ROS [55–58], protection obtained in the presence of taurine also supports oxidative stress involvement in the cytotoxicity mechanism of these NPs in the present model system. In agreement with other studies, all our findings suggest that oxidative stress has an important role in ZnO and CuO NPs-induced cytotoxic effects. Additionally, this protection was also effective against the cytotoxic effect of the mixture of these NPs.

Oxidative stress and ROS have been linked to cell death of neurons in many neurodegenerative conditions [59]. In order to determine the role of possible ROS production in the cytotoxicity mechanisms of these NPs, ROS levels were measured using DCFH-DA assay. In the present study, ROS levels were slightly increased only with the highest concentration (800 µg/ml) of CuO NPs and all concentrations (100:100, 400:400, and 800:800 µg/ml) of the mixture of NPs. These findings could be considered supportive evidence of the protection obtained with NAC and taurine against the cytotoxic effects of these NPs. Similar to our results, increases in ROS levels by CuO NPs were also shown in various cell cultures. For example, Siddiqui et al. [60] reported that 22-nm-sized CuO NPs (2–10 µg/ml) induced the intracellular production of ROS dose-dependently in human hepatocarcinoma cells following 24 h of incubation.

On the other hand, it has been reported that DCF formation was affected in the presence of reducing agents [61]. Therefore, in the present study, due to an interaction possibility, DTPA, NAC, or taurine were not used in DCFH-DA analysis.

Mitochondrial membrane potential and apoptosis levels were also evaluated to understand the underlying mechanisms of cytotoxic effects of ZnO and CuO NPs in SH-SY5Y cells. In this study, the mitochondrial membrane potential of SH-SY5Y cells was found lower than the control group following exposure to the highest concentration (800 µg/ml) of ZnO NPs, and the lowest (100 µg/ ml) concentration of CuO NPs. Actually, decreases were found slight and determined as 10% and 8% for 800 µg/ml ZnO and 100 µg/ml CuO NPs, respectively. Similarly, decreases in MMP levels were reported following ZnO NPs exposure in various cell cultures including HepG2 [62], C3A [63], and retinal ganglion cells [64]. However, Valdiglesias et al. [23] showed that ZnO NPs caused an increase in MMP levels at 20, 30, and 40 µg/ml concentrations following 3 h, and at 30 and 40 µg/ml concentrations following 6 h of incubation in SH-SY5Y cells. On the other hand, similar to our results, Siddiqui et al. [60] reported that CuO NPs had MMP-decreasing effects in HepG2 cells in conditions different from the present study.

It has been known that mitochondrial membrane potential decreases during apoptosis [60, 65]. Caspase 3/7 activity which shows the status of apoptosis was usually increased following exposure to nanoparticles. Likewise, in the present study, these activities were induced following 24 h of exposure to ZnO and CuO NPs at concentrations we have determined according to the results of cytotoxicity tests. In the presence of the mixture of ZnO and CuO NPs, caspase 3/7 activities were also increased. Similar results were also obtained in various cell types including SH-SY5Y cells. For instance, Kim et al. [24] reported that total apoptotic cells (as evaluated alterations of Annexin V and caspase-3/7 activity) were significantly increased at 10–15 µg/mL concentrations of ZnO NPs. Additionally, Song et al. [66] showed that caspase 3/7 activity of astrocyte cells was increased significantly following exposure to 3 and 10 µg/ml concentrations of ZnO NPs at 12 h. It has been reported that higher concentrations of ZnO NPs (15–30 µg/ml) had similar effects on apoptosis in human bone marrow-derived mesenchymal stem cells following 12 and 24 h of incubation [9]. Siddiqui et al. [60] indicated that 10 µg/ml concentration of CuO NPs significantly upregulated apoptotic genes bax and caspase-3 in HepG2 cells following 4 h of incubation. However, in several studies, it has been reported [67, 68] that similar concentrations of the same nanoparticles did not have the same effects on caspase 3/7-mediated apoptosis. This might be due to the differences in culture conditions or cellular environment [66].

In the current study, DTPA alone caused an increase in caspase 3/7 levels; however, it did not reduce ZnO and CuO NP-induced caspase 3/7 levels. On the other hand, we found that NAC or taurine had protective effects. Apoptosis-decreasing effects of NAC or taurine in cell culture systems were also reported in a few studies [69, 70]. For example, Ostrovsky et al. (2009) indicated that due to the ROS quenching effects of NAC, U87 cells protected against ZnO NPs-induced apoptosis.

It has been reported that intracellular calcium levels increased in the presence of nanoparticles [71–73]. However, there was not any study on the effects of CuO NPs on calcium levels in cell culture systems. In the present study, there were decreases in intracellular calcium levels of SH-SY5Y cells following 24 h of incubation of cells with ZnO and CuO NPs individually or as a mixture. DTPA, NAC, or taurine had also similar effects in these levels.

## Conclusions

**Fig. 10:**
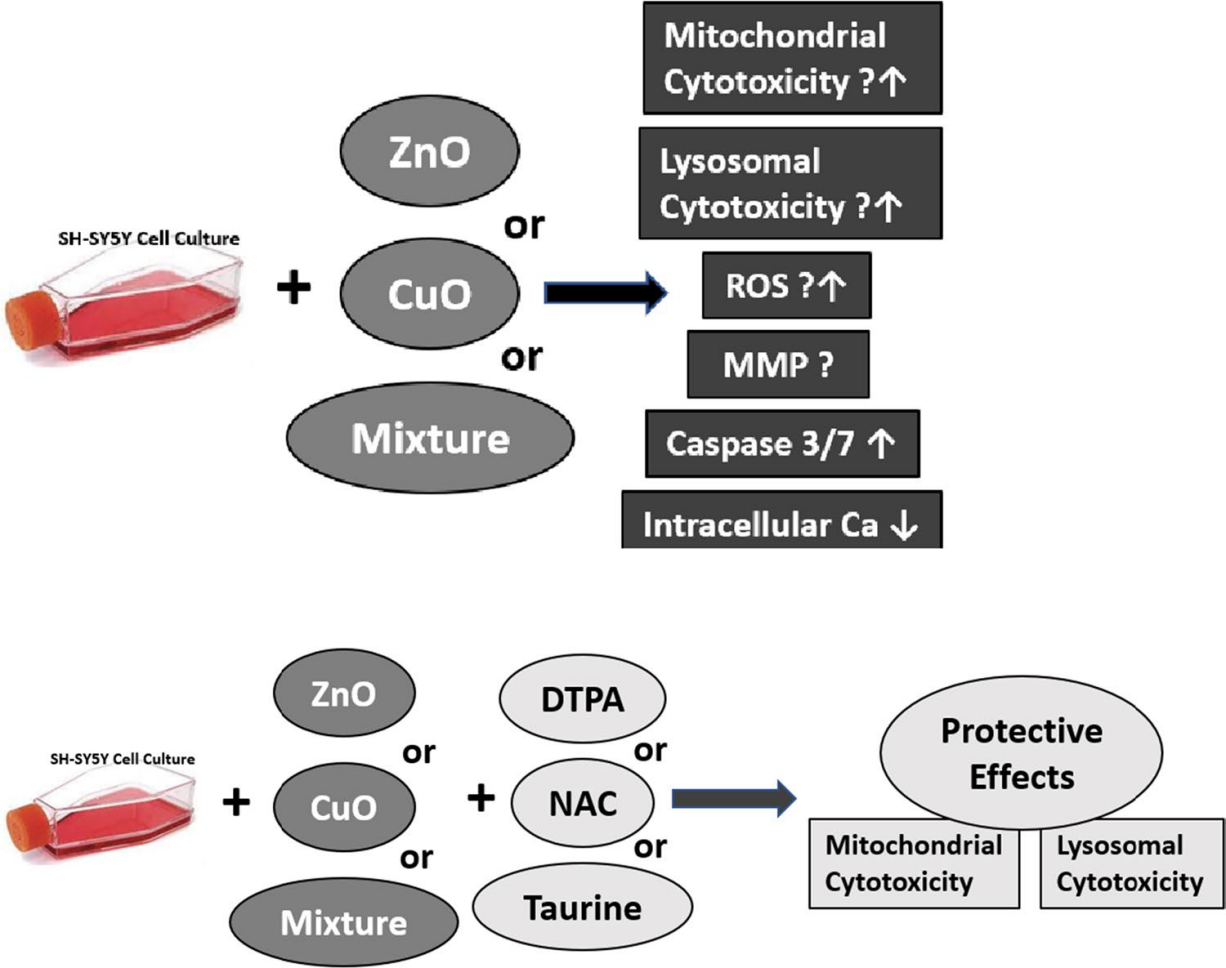
ZnO and CuO NPs induced slight cytotoxic effects either individually or as a mixture in SH-SY5Y cells. Protection noted in the presence of DTPA, NAC, or taurine against cytotoxicity of these NPs. ROS inducing effect of CuO NPs and mixture of two NPs suggest that oxidative stress might be involved in cytotoxicity mechanisms of these NPs

In conclusion, present results indicated that ZnO or/and CuO NPs have slight mitochondrial and lysosomal cytotoxic effects in human neuroblastoma cell lines. Protection obtained against these effects with DTPA suggests that Zn^2+^ and Cu^2+^ ions might have a role in the NPs-induced cytotoxicity. On the other hand, the protective effects provided by NAC and taurine suggest that oxidative stress plays a role in the cytotoxicity mechanism of these NPs. Alterations in the levels of ROS and MMP were also shown in this model system. In addition to protective effects obtained with NAC and taurine in cytotoxicity assays, our results showing ROS inducing potential of CuO NP alone and the mixture of ZnO and CuO NPs might be considered as supportive evidence to the role of ROS in cytotoxicity mechanisms of these NPs (Fig. 10). DTPA, NAC, or taurine had protective effects against apoptosis induced by these NPs. We believe that the data obtained in the current study provide new knowledge about either alone or in mixtures cytotoxic effects of ZnO and CuO NPs. However, additional studies are needed to understand the exact toxicity mechanisms of these NPs.
